# Sexual Debility in Man

**Published:** 1901-11

**Authors:** 


### Books and Magazines.

SEXUAL DEBILITY IN MAN. By F. R. Sturgis, M. D., formerly Clinical Professor of Venereal Diseases in the Medical Department of the University of the City of New York, etc. Pp. 432; price $3. New York: E. B. Treat & Co., 241 and 243 West Twenty-third Street. 1900.

The author of this book has, for many years, devoted his attention to venereal and genito urinary diseases, and is the author of "A Manual of Venereal Diseases," and was one of the authors of "A System of Legal Medicine." In the present volume the author is in full accord with the most advanced ideas in relation to the different phases of this subject. For instance, in the chapter on masturbation he has combatted the old and time honored belief that indulgence in this habit is the necessary prelude to both physical and mental degeneration. He does not gloss over the dangers which may, under certain conditions, result from the habit, but he has attempted to point out the folly of the hysterical denunciations that have been heapet upon it by pseudo-philanthropists and ignorant medical men. He believes that castration in the case of masturbating lunatics, under certain circumstances, is justifiable and proper.

The book is divided into twelve chapters, and is devoted to the following general subjects: the anatomy and physiology of the sexual organs, male and female; the etiology and pathology of masturbation and onanism with treatment; spermatorrhea, polutions, sexual impotence and sterility, considering the physical, psychical and reflex causes, with treatment.

The book embodies advanced and reliable views, and will no doubt receive much attention. T. J. B.

